# Endothelial Toll-like receptor 4 is required for microglia activation in the murine retina after systemic lipopolysaccharide exposure

**DOI:** 10.1186/s12974-023-02712-1

**Published:** 2023-02-04

**Authors:** Ioanna Tsioti, Beatrice L. Steiner, Pascal Escher, Martin S. Zinkernagel, Peter M. Benz, Despina Kokona

**Affiliations:** 1 Department of Ophthalmology, Inselspital, Bern University Hospital, University of Bern, Bern, Switzerland; 2 Department of BioMedical Research, University of Bern, Bern, Switzerland; 3 Department of CardioMetabolic Diseases Research, Boehringer Ingelheim Pharma GmbH & Co. KG, Biberach, Germany

**Keywords:** Retina, Microglia, Lipopolysaccharide, Toll-like receptor 4, Endothelial cells, Monocyte-derived macrophages

## Abstract

**Background:**

Clustering of microglia around the vasculature has been reported in the retina and the brain after systemic administration of lipopolysaccharides (LPS) in mice. LPS acts via activation of Toll-like receptor 4 (TRL4), which is expressed in several cell types including microglia, monocytes and vascular endothelial cells. The purpose of this study was to investigate the effect of systemic LPS in the pigmented mouse retina and the involvement of endothelial TLR4 in LPS-induced retinal microglia activation.

**Methods:**

C57BL/6J, conditional knockout mice that lack *Tlr4* expression selectively on endothelial cells (Tek^Cre−pos^Tlr4^loxP/loxP^) and Tek^Cre−neg^Tlr4^loxP/loxP^ mice were used. The mice were injected with 1 mg/kg LPS via the tail vein once per day for a total of 4 days. Prior to initiation of LPS injections and approximately 5 h after the last injection, in vivo imaging using fluorescein angiography and spectral-domain optical coherence tomography was performed. Immunohistochemistry, flow cytometry, electroretinography and transmission electron microscopy were utilized to investigate the role of endothelial TLR4 in LPS-induced microglia activation and retinal function.

**Results:**

Activation of microglia, infiltration of monocyte-derived macrophages, impaired ribbon synapse organization and retinal dysfunction were observed after the LPS exposure in C57BL/6J and Tek^Cre−neg^Tlr4^loxP/loxP^ mice. None of these effects were observed in the retinas of conditional *Tlr4* knockout mice after the LPS challenge.

**Conclusions:**

The findings of the present study suggest that systemic LPS exposure can have detrimental effects in the healthy retina and that TLR4 expressed on endothelial cells is essential for retinal microglia activation and retinal dysfunction upon systemic LPS challenge. This important finding provides new insights into the role of microglia–endothelial cell interaction in inflammatory retinal disease.

**Supplementary Information:**

The online version contains supplementary material available at 10.1186/s12974-023-02712-1.

## Background

Toll-like receptors (TLRs) are a subfamily of pattern recognition receptors (PRRs) essential for the initiation of inflammatory responses. Among 12 different TLRs that have been identified in mice, TLR4 is a main contributor to neuronal cell death, ischemic brain injury, and breakdown of the blood brain barrier [1–4]. In the retina, TLR4 is mainly expressed by microglia cells [5], Müller cells [6], retinal pigment epithelial cells (RPEs) [7, 8], and vascular endothelial cells [9]. TLR4 is primarily activated by lipopolysaccharides (LPS); a bacterial endotoxin exposed on Gram-negative bacterial walls, allowing host cells to react to bacterial invasions by initiating rapid and robust inflammatory responses [10–12].

LPS is the most commonly used molecule to trigger microglia and macrophages activation both in vivo and in vitro. In the central nervous system (CNS), microglia are involved in neuronal survival and function, they are the first responders to invading pathogens, and they actively participate in the inflammatory response together with monocyte-derived macrophages and other immune cells invading from the circulation after infection or injury [13]. Microglia activation in the retina after systemic LPS exposure has been reported, and was correlated with exacerbation of retinal inflammation, increased photoreceptor death and worsening of retinal function in a genetic model of retinal degeneration, the RHO-P23H rat [14]. Moreover, LPS-induced systemic inflammation in P4 mouse pups led to abnormalities in the development of retinal vasculature and permanent impairment of retinal function, both associated with microglia activation [15]. On the other hand, LPS preconditioning has been shown to act in a neuroprotective manner in retina and brain disease [16–18]. In the healthy CNS though, repetitive systemic challenges with LPS lead to widespread activation of microglia both in the retina and the brain [16, 19, 20] and we have recently shown that they can also lead to blood retinal barrier (BRB) breakdown in albino mice [19]. An intact BRB preserves retinal homeostasis acting as a physical barrier against the entry of molecules and pathogens from the periphery that could otherwise disturb retinal physiology [21]. The involvement of TLR4 in blood brain barrier (BBB) disruption has been suggested in rodents [4, 22, 23], while LPS-induced BRB breakdown suggests a role of TLR4 in BRB breakdown as well [19].

RPE cells together with the underlying Bruch’s membrane and the fenestrated choriocapillaris form the outer BRB. The inner BRB is formed by endothelial cells’ interactions with pericytes, neurons, astrocytes, microglia and Müller cells, and insulates the inner retina from the deep, intermediate, and superficial vascular plexus. Endothelial cells are connected with tight junctions and create a tight monolayer that prevents the entry of foreign material into the retina, contributing to immune privilege [24]. These cells, actively participate in immune regulation during inflammation, by upregulating the expression of cell adhesion molecules and chemokines that facilitates the rolling and influx of leukocytes into the CNS (for a recent review see Ref. [25]).

Systemic LPS triggers activation of both endothelial cells and microglia in the CNS [26–28]. However, whether LPS-induced microglia activation precedes endothelial activation or vice versa is unclear. For instance, vascular damage caused by methamphetamine or thiamine deficiency has been shown to attract microglia towards the affected vasculature, indicating that vascular damage is the event that triggers microglia migration and activation in an effort to repair the affected vessels [29, 30]. However, disruption of the BBB by activated microglia has been proposed in vitro [31]. Previous studies have shown that brain neurodegeneration and vascular damage are the cause and not the consequence of microglia activation [32, 33]. Older studies also suggested that activation of microglia is mostly beneficial and could rarely initiate neurodegenerative cascades in the brain [34]. We have previously shown that LPS-induced BRB disruption correlated with monocyte-derived macrophages influx and clustering of microglia/macrophages around the retinal endothelium in Balb/c mice, indicating microglia/macrophage–endothelium interactions following systemic LPS exposure [19]. The influx of monocyte-derived macrophages into the central nervous system upon BRB and BBB breakdown has been described in several disease models (for a recent review see Ref. [35]). These cells express TLR4 that allows their direct activation by LPS [36] and their interaction with activated endothelial cells plays a major role in their ability to penetrate brain vessels [37, 38].

In a previous study using bone marrow chimera mice, researchers have shown that TLR4 expressed on endothelial cells and microglia, rather than on cells originating in the blood, is required for systemic LPS-induced microglia activation in a murine brain injury model [16]. Based on that study and since endothelial cells bear TLR4 and are in direct contact with blood, acting as a barrier that insulates the retina from the periphery, we hypothesized that systemic LPS would first bind to endothelial TLR4 before directly activating microglia TLR4. To test our hypothesis, we employed wild-type C57BL/6J mice and generated mice that lack *Tlr4* expression selectively on endothelial cells. First, we investigated the effect of systemic LPS exposure on the wild-type C57BL/6J pigmented retina and on the Tek^Cre−neg^Tlr4^loxP/loxP^ retina, and secondly, the impact of endothelial *Tlr4* depletion on LPS effects. Our data support the hypothesis that upon systemic LPS exposure, activation of TLR4 located on endothelial cells is essential for subsequent microglia activation, monocyte-derived macrophages influx, microglia/macrophage interactions with endothelial cells and functional impairment.

## Materials and methods

### Animals

This study was approved by the local Animal Ethics Committee (Veterinärdienst des Kantons Bern: BE53/17 and BE3/2021) and conformed to the Association for Research in Vision and Ophthalmology Statement for the Use of Animals in Ophthalmic and Vision Research. Adult 12–20 weeks male and female C57BL/6J mice, conditional knockout mice that are deficient for *Tlr4* expression in endothelial cells (Tek^Cre−pos^Tlr4^loxP/loxP^ mice) and Tek^Cre−neg^Tlr4^loxP/loxP^ mice were used in this study. The conditional knockout mice were generated by breeding B6.Cg-Tg(Tek-cre)1Ywa/J mice that express Cre recombinase under the direction of the receptor tyrosine kinase Tek (*Tie2*) promoter/enhancer, which has been shown to provide uniform expression in endothelial cells [39], with B6(Cg)-Tlr4^tm1.1Karp^/J mice (The Jackson Laboratory strain #024872), which possess loxP sites flanking exon 3 of the *Tlr4* gene. Tek^Cre^tdTomato mice were used to confirm the lack of tdTomato expression in Iba-1-positive cells (Iba-1^pos^). Animals were housed under controlled temperature and humidity conditions in ventilated cages with a 12-h light–dark cycle. All mice had free access to food and water.

### Systemic LPS challenge and in vivo imaging

Mice were injected via the tail vein once per day for a total of 4 days with 1 mg/kg LPS from *Escherichia coli* serotype O111:B4 (Cat # L2630; Sigma-Aldrich, Buchs, Switzerland) as previously described [19]. Fluorescein angiography (FA) and spectral-domain optical coherence tomography (SD-OCT) were used for the visualization of retinal vasculature and retinal structure, before the first LPS challenge and approximately 5 h after the last LPS challenge, using a Heidelberg Spectralis system (Heidelberg Spectralis HRA 2; Heidelberg Engineering GmbH, Heidelberg, Germany) as described elsewhere [19]. Before and after in vivo imaging, the mice were anesthetized and the anesthesia was reversed, respectively, as described elsewhere [19]. At the end of the experiments, mice were euthanized with CO_2_ inhalation followed by decapitation.

### Flow cytometry analysis (FACS)

Naïve and LPS-challenged C57BL6/J and Tek^Cre−pos^Tlr4^loxP/loxP^ mice were euthanized approximately 16 h after the last LPS injection and their retinas were prepared for flow cytometry analysis as described elsewhere [19]. Both retinas of each mouse were treated as one sample. Dead cells stained with Zombie Green™ Fixable Viability Kit (1:800; Biolegend, San Diego, CA, USA; Cat # 423111) were excluded from the analysis. Extracellular markers against the leukocyte common antigen CD45 (30-F11; 1:400; allophycocyanin-Cy7/APC-Cy7; Cat # 103115) and the CD11b antigen-like family member B (M1/70; 1:200; allophycocyanin/APC; Cat # 101212) were used for the labeling of microglia as CD11b positive and CD45 low or negative (CD11b^pos^CD45^low/neg^) and macrophages as CD11b positive and CD45 high (CD11b^pos^CD45^hi^) expressing cells [40–43]. Fluorescein isothiocyanate (FITC) conjugated antibodies against CD19 (6D5; 1:200; Cat #115505), CD3 (17A2; 1:200; Cat #100203), NK1.1 (PK136; 1:200; Cat #108705) and Ly6G (1A8; 1:200; Cat #127605) were used for the exclusion of B-lymphocytes, T cells, natural killer cells and granulocytes, respectively. To test the selective ablation of TLR4 from endothelial cells in the conditional knockout mice, we used naïve retinas from Tek^Cre−pos^Tlr4^loxP/loxP^ and Tek^Cre−neg^Tlr4^loxP/loxP^ mice. Single-cell suspensions were stained with a TIE2 antibody (TEK4; 1:100; allophycocyanin/APC; Cat # 124009) and a primary mouse monoclonal TLR4 antibody (76B357.1; 1:400; Novus Biologicals, Littleton, CO, USA; Cat # NB100-56566SS), followed by incubation with a secondary goat F(ab′)2 anti-mouse IgG H&L (phycoerythrin, PE) pre-adsorbed antibody (1:100; Cat # ab7002; Abcam, Cambridge, United Kingdom). DAPI (1:800; Thermo Fisher Scientific, Waltham, MA, USA Cat # 62248) was used for exclusion of dead cells [44]. Flow cytometry antibodies were purchased from Biolegend (San Diego, CA, USA) unless otherwise indicated above.

### Immunohistochemistry

One eye per mouse was used for immunohistochemistry and one for retinal whole mounts. For immunohistochemistry, eyes were enucleated and fixed in 4% paraformaldehyde in 1× PBS (pH 7.4) overnight and embedded in paraffin. Slides with 5-μm sections were deparaffinized, rehydrated, and incubated for 15 min in Tris–EDTA buffer (pH 9.0) at 95 °C. Slices were blocked for 30 min with blocking buffer [5% normal goat serum (NGS, Lucerna-Chem AG, Luzern, Switzerland) in 0.2% Triton X-100 in 1× PBS] and incubated with the following primary antibodies: a rabbit polyclonal antibody against ionized calcium binding adapter molecule 1 (Iba-1, 1:1000; Cat # 0162-0001, FUJIFILM Wako Shibayagi, Japan), a mouse monoclonal antibody against c-terminal binding protein-2 (CtBP2, 1:250; Cat # 612044, BD Biosciences, Basel, Switzerland), and a rat monoclonal antibody against intercellular adhesion molecule 1 (ICAM-1, 1:200; Cat # ab119871, Abcam, Cambridge, United Kingdom). The sections were incubated with the primary antibodies overnight at 4 °C. All antibodies were diluted in blocking buffer. The slides were washed in 1× PBS and incubated with the secondary antibodies goat anti-rabbit Alexa Fluor 594 conjugate (1:1000; Cat # A11012, ThermoFisher scientific, Waltham, MA, USA), goat anti-mouse F(ab′)2 IgG (H + L) cross-adsorbed secondary antibody Alexa Fluor 488 conjugate (1:1000; Cat # A48286, ThermoFisher scientific, Waltham, MA, USA) and/or a goat anti-rat IgG (H + L) cross-adsorbed secondary antibody, Alexa Fluor™ 488 (1:1000; Cat # A-11006, ThermoFisher scientific, Waltham, MA,) for 1 h at RT. Slides were washed again and mounted in mounting medium with DAPI (Vector Laboratories, Reactolab SA, Servion, Switzerland).

### Retinal whole mounts

For retinal whole mounts, eyes were isolated and processed as described elsewhere [45]. Retinas were incubated with a primary antibody against Iba-1 diluted in blocking buffer (1:500; anti-rabbit Cat #019-19741, FUJIFILM Wako Shibayagi, Japan) overnight at 4 °C, for labeling of microglia/macrophages. Samples were washed in 0.2% TritonX-100 in 1× PBS and incubated with goat anti-rabbit Alexa Fluor 594 conjugate (1:200; Cat # A11012, ThermoFisher scientific, Waltham, MA, USA) for 2 h at RT. Retinas were flattened as described elsewhere [45].

### Microscopy

Retinal paraffin sections stained for Iba-1 and ICAM-1 were examined under a fluorescence microscope (Nikon Eclipse 80i microscope; Nikon, Tokyo, Japan). An inverted Zeiss LSM 710 fluorescence confocal microscope (Carl Zeiss, Oberkochen, Germany) was utilized for the imaging of the retinal whole mounts and for the CtBP2 staining. For Iba-1 staining in retinal whole mounts, Z-stacks with 0.85 μm interval were obtained using Zen system 2011 software (Carl Zeiss) with a 20× lens (Plan-Apochromat 20×/0.8 M27/a = 0.55 mm). For the CtBP2 staining, Z-stacks with 0.5 μm interval were obtained with a 100× lens (Plan-APOCHROMAT 100×/1.4 Oil Ph3). Figures were finalized in Coreldraw 2021.5 (Corel Corporation, Ottawa, ON, Canada).

### Quantification of Iba-1^pos^ cells morphology

For the quantification of branch length and endpoints per Iba-1^pos^ cell, z-stacks of retinal whole mounts were exported as individual images using the ZEN system 2011 software (Carl Zeiss, Oberkochen, Germany). Individual images corresponding to the area between the ganglion cell layer and the inner nuclear layer (GCL-INL) or outer plexiform layer (OPL) were combined for the generation of 2 new z-stacks. Z-stacks were z-projected and converted to grayscale. Skeleton analysis was performed in the z-stacks derived from the OPL using the ImageJ 1.52 h software according to a published protocol [46]. The total branch length and the total number of endpoints per image were divided by the number of Iba-1^pos^ cells in each image. For measurements of Iba-1^pos^ cells volume, ImageJ 1.52 h software was used. Image threshold was adjusted in z-stacks containing the retinal thickness from the GCL to the OPL, and the Iba-1 occupied volume was measured using the “Measure stack” plugin. Iba-1 volume is expressed as a percentage of the total z-stack volume.

### Retinal vasodilation measurements

Retinal vasodilation was measured in SD-OCT b-scans, measuring the diameter of the veins in the different layers of the retina, using the Heyex software version 1.9.17.0 (Heidelberg Engineering Inc., Franklin, USA). Three different veins from 6 b-scans were measured and averaged per eye.

### Electroretinography (ERG) recordings

Retinal function of LPS-challenged mice was recorded at baseline and 4 days after the first LPS challenge, using electroretinography (ERG). The animals were recorded in photopic and scotopic conditions to measure the function of cone and rod photoreceptors, respectively, upon the LPS stimuli. To achieve cone saturation, mice were dark adapted overnight before all measurements. Mice were anesthetized and waked up as previously described [47]. The pupils were dilated with tropicamide 0.5% and phenylephrine 2.5% eyedrops. A drop of Viscotears Liquid Gel was placed on the eyes to avoid mydriasis. Two reference electrodes with platinum needles were placed below the eyes and one ground electrode was placed above the tail. Gold rings contact electrodes were placed on the eyes of the animals and the animals were positioned on a custom-made platform. For measurements of *a*- and *b*-waves in scotopic conditions, ten different increasing luminances from − 4 to 1.5 log were used. A light adaptation period of 10 min was followed by eight different elevating intensities from − 2.0 to 1.5 log to measure both waves in photopic conditions. For the quantification of *b*-waves in both conditions, a filter to remove the oscillatory potentials was applied.

### Transmission electron microscopy (TEM)

Mice were deeply anesthetized with 150 mg/kg pentobarbital (Esconarkon, 300 mg/ml, Streuli Pharma AG, Uznach, Switzerland) diluted in 0.9% NaCl. Under deep anesthesia, mice were transcardially perfused with 50 ml 1× PBS supplemented with 10 U/ml heparin (in 0.9% NaCl; Inselspital, Bern, Switzerland), followed by 50 ml of Karnovsky solution (2.5% glutaraldehyde, and 2% paraformaldehyde in 0.1 M Na-cacodylate buffer pH 7.39). Mouse eyes were isolated and fixed with Karnovsky solution for at least 24 h before being further processed. Samples were washed with 0.1 M Na-cacodylate buffer (Merck, Darmstadt, Germany) and postfixed with 1% OsO4 (Electron Microscopy Sciences, Hatfield, USA) in 0.1 M Na-cacodylate buffer at 4 °C for 2 h. After washing, the samples were dehydrated in 70, 80, 96% and 100% ethanol (Alcosuisse, Switzerland), followed by acetone (Merck, Darmstadt, Germany), and finally samples were immersed in acetone–Epon (1:1) overnight. Samples were embedded in Epon (Sigma-Aldrich, Buchs, Switzerland) and left to harden at 60 °C for 5 days. Sections were produced with an ultramicrotome UC6 (Leica Microsystems, Vienna, Austria), first semithin sections (1 μm) for light microscopy which were stained with a solution of 0.5% toluidine blue O (Merck, Darmstadt, Germany) and then ultrathin sections (75 nm) for electron microscopy. The sections, mounted on Formvar® (Ted Pella Inc., USA) coated single slot copper grids, were stained with Uranyless (Electron Microscopy Sciences, Hatfield, USA) and lead citrate (Leica Microsystems, Vienna, Austria) with an Ultrostainer (Leica Microsystems, Vienna, Austria). Sections were examined with a transmission electron microscope (Tecnai Spirit, FEI, Brno, Czech Republic) equipped with a digital camera (Veleta, Olympus, Soft Imaging System, Münster, Germany).

### Statistics

For the statistical analysis, GraphPad Prism software version 8.0 was used (GraphPad Software, Inc., San Diego, CA). Statistically significant differences between naïve and LPS groups were determined using unpaired two-tailed t-tests except for the vasodilation data, where paired two-tailed *t*-test was used. ERG data were compared with repeated measures 2-way ANOVA followed by Sidak’s post hoc analysis. All data are expressed as means ± SD. *P* values < 0.05 were considered statistically significant. Single dots in bar graphs represent individual samples (2 retinas from a single mouse for the FACS data and 1 eye per mouse for the rest of the data).

## Results

### Endothelial TLR4 depletion in the Tek^Cre−pos^Tlr4^loxP/loxP^ mouse retina

**Fig. 1:**
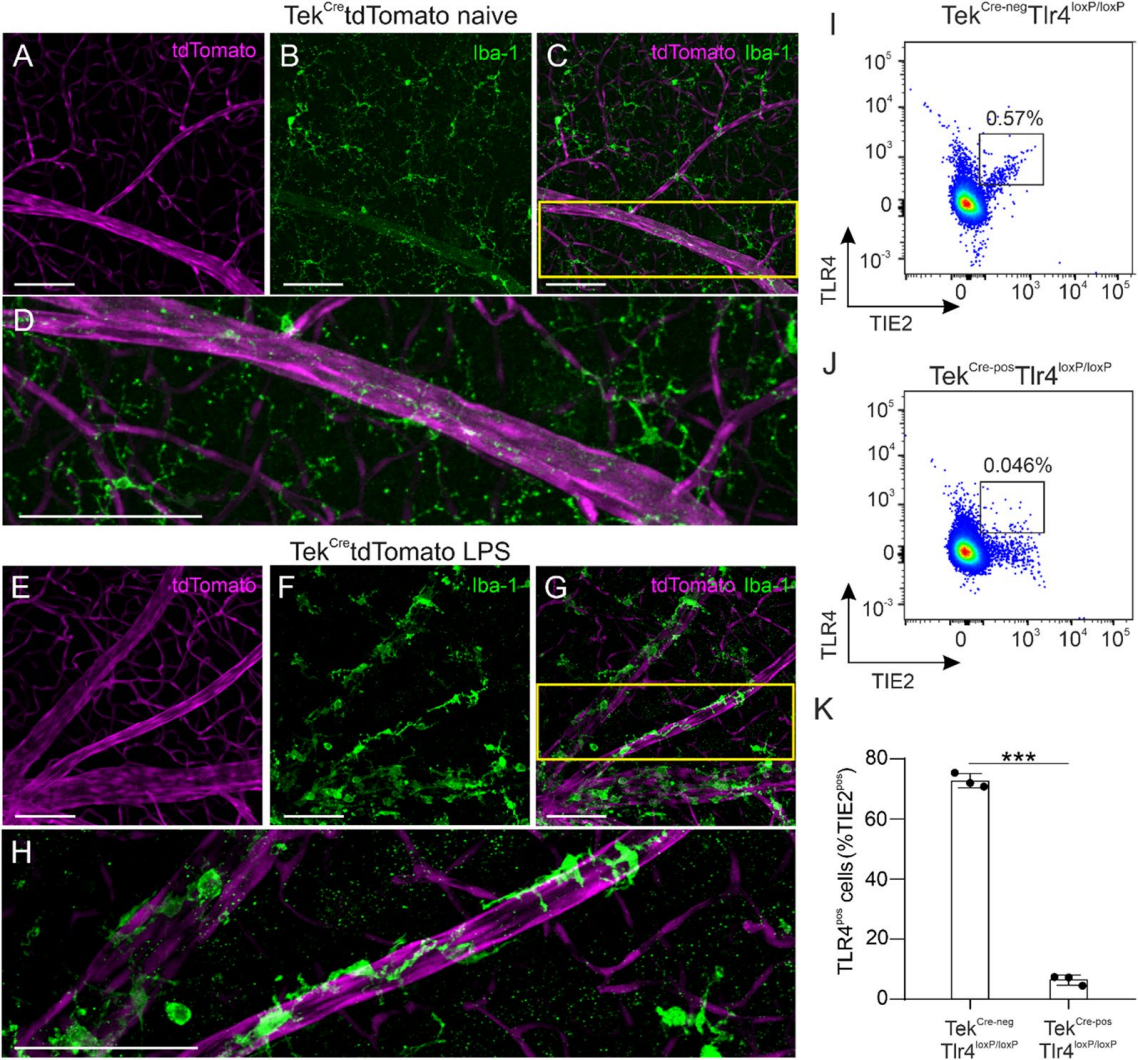
Cre-mediated recombination selectively depletes TLR4 on endothelial cells. Representative retinal whole mounts of Tek^Cre^tdTomato mice stained with Iba-1 (green) in naïve mice (**A**–**C**) and LPS-challenged mice (**E**–**G**). tdTomato (magenta) was selectively expressed in retinal blood vessels and no co-localization with Iba-1 was detected. The yellow squares in C and G are magnified in **D** and **H**, respectively. Scale bars: 100 μm. Representative dot plots of single, live, TIE2^pos^TLR4^pos^ cells in the retinas of naïve Tek^Cre−neg^Tlr4^loxP/loxP^ and Tek^Cre−pos^Tlr4^loxP/loxP^ mice (**I**, **J**). The majority of TIE2^pos^ cells (72.7%) in the Tek^Cre−neg^Tlr4^loxP/loxP^ retinas express TLR4, while in the retinas of Tek^Cre−pos^Tlr4^loxP/loxP^ mice only 6.4% of TIE2^pos^ cells are TLR4^pos^ (**K**; *n* = 3 mice per group). The data were analyzed with 2-tailed unpaired *t*-test, ***p < 0.001)

Mice that lack *Tlr4* expression selectively in endothelial cells (Tek^Cre−pos^Tlr4^loxP/loxP^) were generated to investigate the role of endothelial TLR4 on LPS actions in the retina. Since expression of *Tie2* has been reported in microglia and macrophages as well [48], we used Tek^Cre^tdTomato mice to evaluate the specificity of Cre-mediated recombination in endothelial cells. In Iba-1-stained retinal whole mounts of naïve (Fig. 1A–D) and LPS-challenged (Fig. 1E–H) Tek^Cre^tdTomato mice, tdTomato was expressed on retinal vessels and was not co-localized with the microglia/macrophage marker Iba-1. Ablation of TLR4 expression from endothelial cells was substantiated by FACS analysis. The number of TLR4^pos^TIE2^pos^ cells was drastically reduced in the Tek^Cre−pos^Tlr4^loxP/loxP^ mouse retinas compared to their Tek^Cre−neg^Tlr4^loxP/loxP^ littermate controls (Fig. 1I–K). These observations substantiate that Cre recombinase is selectively expressed in endothelial cells and Cre-mediated recombination selectively depletes endothelial TLR4 in the retinas of Tek^Cre−pos^Tlr4^loxP/loxP^ mice.

### Effect of systemic LPS exposure on retinal microglia activation and retinal vasodilation in the presence or absence of endothelial TLR4

**Fig. 2:**
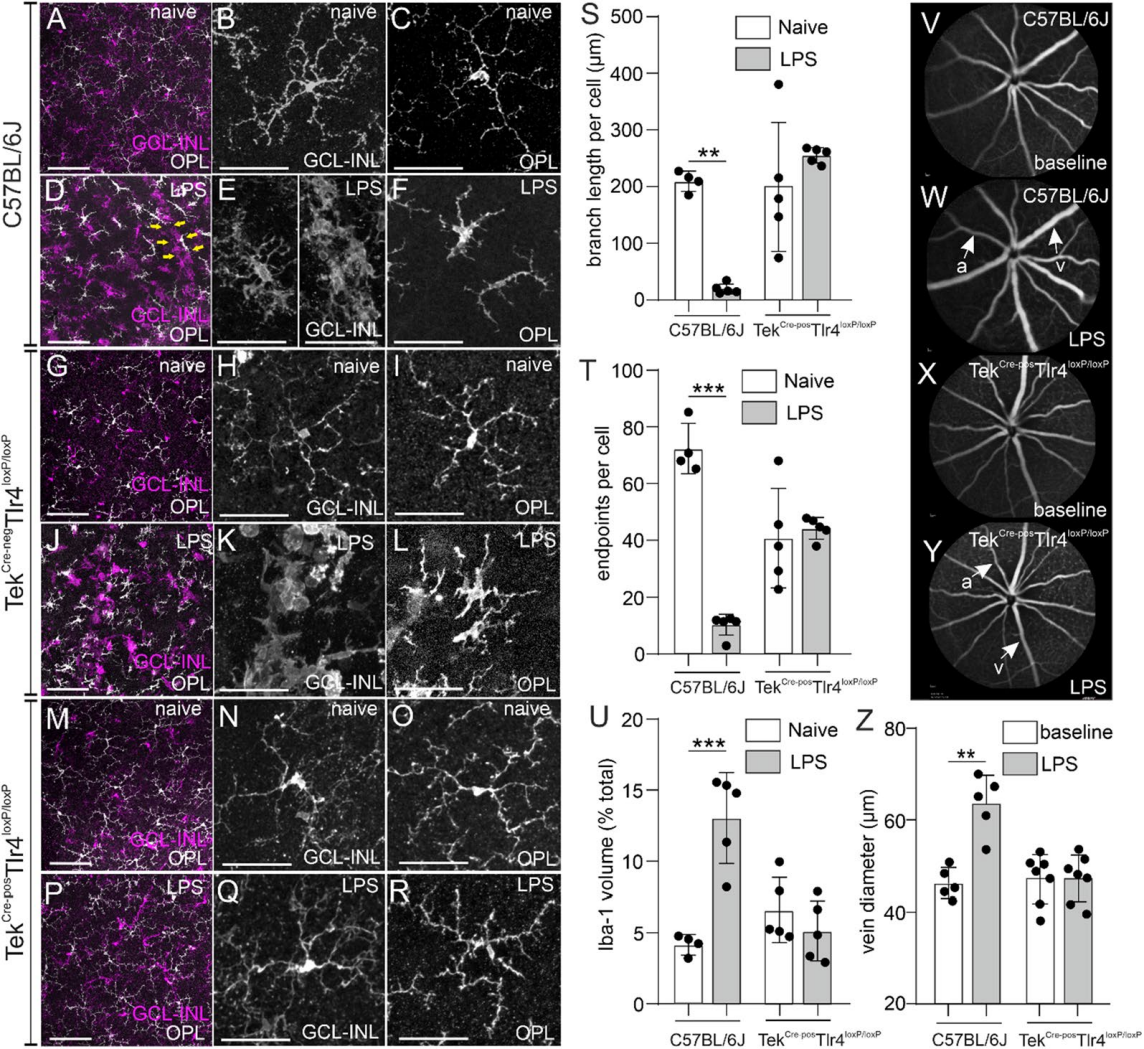
Systemic LPS fails to trigger microglia activation and retinal vasodilation in endothelial *Tlr4*-deficient mice. Representative images of Iba-1-stained retinal whole mounts in naïve (**A**, **G**, **M**) and LPS-challenged (**D**, **J**, **P**) C57BL/6J, Tek^Cre−neg^Tlr4^loxP/loxP^ and Tek^Cre−pos^Tlr4^loxP/loxP^ mice, respectively. Iba-1^pos^ cells located in the GCL-INL and the OPL are shown in magenta and white, respectively. Yellow arrows in D indicate the clustering of Iba-1^pos^ cells around a retinal vessel. In naïve C57BL/6J mice, microglia cells have a ramified morphology throughout the retina (**B**, **C**). After the LPS challenge, Iba-1^pos^ cells adopt a bushy or amoebic morphology and accumulate around retinal blood vessels in the GCL-INL (**E**). In the OPL, these cells have an amoebic or rod-like shape (**F**). The morphology of Iba-1^pos^ cells before or after the LPS challenge in Tek^Cre−neg^Tlr4^loxP/loxP^ mice was comparable to C57BL/6J mice (**H**, **I**, **K**, **L**). Individual Iba-1^pos^ cells of Tek^Cre−pos^Tlr4^loxP/loxP^ mice had a ramified morphology both in the naïve and the LPS-challenged group (**N**, **O**, **Q**, **R**). Quantification of average branch length (**S**; *n* = 4–5 mice per group), number of endpoints per cell (**T**; *n* = 4–5 mice per group) and Iba-1 occupied volume (**U**; *n* = 4–5 mice per group) on images obtained from retinal whole mounts. Reduced branch length, less endpoints per cell and increased Iba-1 occupied volume were detected in the C57BL/6J but not the endothelial *Tlr4* knockout mice. Representative images of fluorescein angiographs at baseline (**V**, **X**) and after the LPS challenge (**W**, **Y**) in C57BL/6J and Tek^Cre−pos^Tlr4^loxP/loxP^ mice, respectively and quantification of vein dilation (**Z**; *n* = 5–7 mice per group). Vein diameter was significantly increased in LPS-challenged C57BL/6J mice compared to baseline, but not in Tek^Cre−pos^Tlr4^loxP/loxP^ mice. The quantification data are reported as mean ± SD. The data were analyzed separately for each genotype with 2-tailed unpaired *t*-test (**S**–**U**) or 2-tailed paired *t*-test (**Z**). **p < 0.01, ***p < 0.001. *a* Artery; *v* vein; *GCL-INL* ganglion cell layer-inner nuclear layer; *OPL* outer plexiform layer. Scale bars: left panel: 100 μm; middle and right panels: 50 μm

In naïve C57BL/6J retinas, morphology of Iba-1^pos^ cells was typical for “resting” microglia with small soma and long extended processes (Fig. 2A–C). After the LPS exposure, hypertrophic and bushy Iba-1^pos^ cells, with swollen cell bodies and poorly ramified processes were present in the GCL-INL, while clusters of amoebic cells were found around retinal vessels (Fig. 2D, E), most probably representing microglia, perivascular macrophages and monocyte-derived macrophages invading from the periphery. In the OPL of LPS-challenged mice, the morphology of these cells was either amoebic or rod-like with a thick soma and polarized processes (Fig. 2F). The morphology of microglia from Tek^Cre−neg^Tlr4^loxP/loxP^ mice after the LPS challenge was comparable to the C57BL/6J LPS-challenged mice (Fig. 2G–L).In the Tek^Cre−pos^Tlr4^loxP/loxP^ retinas, microglia cells had a typical resting morphology both in naïve and in LPS-challenged mice (Fig. 2M–R). In the OPL, the branch length of Iba-1^pos^ cells and number of endpoints per cell were reduced after the LPS exposure in C57BL/6J retinas indicating cell activation, but not in Tek^Cre−pos^Tlr4^loxP/loxP^ retinas (Fig. 2S, T). Since identification of individual Iba-1^pos^ cells in the GCL-INL was not possible due to cell clustering, we were unable to perform these analyses for those cells. The volume occupied by Iba-1, expressed as a percentage of the total z-stack volume, was also increased in the C57BL/6J LPS-challenged mice but was not affected by the LPS exposure in Tek^Cre−pos^Tlr4^loxP/loxP^ mice (Fig. 2U). These changes were accompanied by significant dilation of retinal veins in C57BL/6J LPS-challenged mice, while no statistically significant changes were observed in vein diameter after the LPS exposure in Tek^Cre−pos^Tlr4^loxP/loxP^ mice (Fig. 2V–Z).

### Effect of systemic LPS exposure on retinal microglia and monocyte-derived macrophage numbers in the presence or absence of endothelial TLR4

**Fig. 3:**
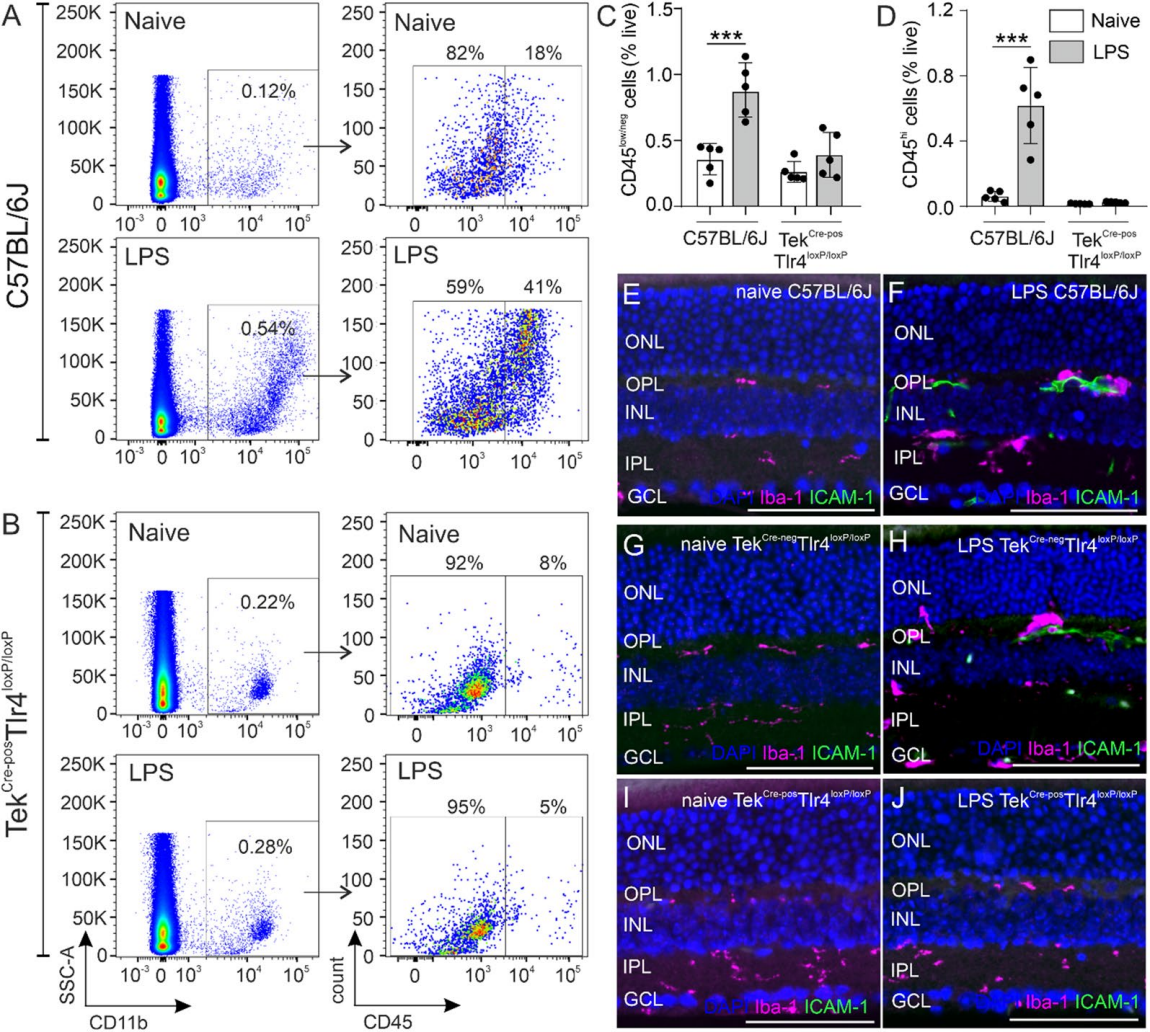
Effect of LPS on microglia/macrophage numbers in the presence or absence of endothelial *Tlr4*. Representative dot plots of CD11b^pos^ populations (**A**, **B** left panels) gated as CD11b^pos^CD45^low/neg^ microglia and CD11b^pos^CD45^hi^ monocyte-derived macrophages (**A**, **B** right panels) in naïve and LPS-challenged C57BL/6J and Tek^Cre−pos^Tlr4^loxP/loxP^ mice, respectively. Quantification of CD45^low/neg^ microglia and CD45^hi^ macrophages expressed as a percentage of the total number of live cells in the samples (**C**, **D**) revealed an increase of both populations after the LPS challenge in C57BL/6J mice but not in Tek^Cre−pos^Tlr4^loxP/loxP^ mice (*n* = 5 mice per genotype and experimental group). The data were analyzed separately for each genotype with 2-tailed unpaired *t*-test (***p < 0.001). Representative images of retinal sections stained with Iba-1 and ICAM-1 in naïve (**E**, **G**, **I**) and LPS-challenged (**F**, **H**, **J**) C57BL/6J, Tek^Cre−neg^Tlr4^loxP/loxP^ and Tek^Cre−pos^Tlr4^loxP/loxP^ mice, respectively. ICAM-1 immunoreactivity was detected in LPS-challenged C57BL/6J and Tek^Cre−neg^Tlr4^loxP/loxP^ mice but not in Tek^Cre−pos^Tlr4^loxP/loxP^ mice. Scale bars: 200 μm. *GCL* Ganglion cells layer; *INL* inner nuclear layer; *IPL* inner plexiform layer; *ONL* outer nuclear layer; *OPL* outer plexiform layer

Flow cytometry was performed in naïve and LPS-challenged C57BL/6J and Tek^Cre−pos^Tlr4^loxP/loxP^ mouse retinas. After exclusion of doublets and dead/FITC-expressing cells (Additional file 1: Fig. S1), the cells were gated based on the expression of CD11 antigen-like family member B (CD11b; also called integrin α-M), known to be expressed by microglia and cells of myeloid lineage [42, 43]. CD11b^pos^ cells were further gated for the expression of the leukocyte common antigen (L-CA) CD45 (also called receptor-type tyrosine-protein phosphatase C) to distinguish between microglia (low expression) and macrophages (high expression) [40–42] (Fig. 3A, B). The total number of microglia and macrophages in the LPS-challenged C57BL/6J retinas was significantly increased, reaching 2.4 and 5.7-fold of the naïve levels, respectively (Fig. 3A right panel, C, D). In the Tek^Cre−pos^Tlr4^loxP/loxP^ retinas, the numbers of microglia and macrophages were comparable between the naïve and LPS-challenged mice (Fig. 3B right panel, C, D). The increase in C57BL/6J microglia/macrophage numbers by LPS was accompanied by expression of ICAM-1 in the outer boundary of the OPL and the inner boundary of the inner plexiform layer (IPL), corresponding to the deep and the intermediate vascular plexus, respectively (Fig. 3F). ICAM-1 expression was found in close proximity to Iba-1^pos^ cells suggesting transmigration of leukocytes into the retina [49]. The same expression of ICAM-1 in proximity to Iba-1^pos^ cells was detected in the retinas of Tek^Cre−neg^Tlr4^loxP/loxP^ mice (Fig. 3H). We did not detect ICAM-1 expression in the retinas of naïve C57BL/6J (Fig. 3E) and Tek^Cre−neg^Tlr4^loxP/loxP^ mice (Fig. 3G); or in the retinas of Tek^Cre−pos^Tlr4^loxP/loxP^ mice neither before (Fig. 3I) nor after the LPS challenge (Fig. 3J).

### Effect of systemic LPS exposure on retinal function in the presence or absence of endothelial TLR4

**Fig. 4:**
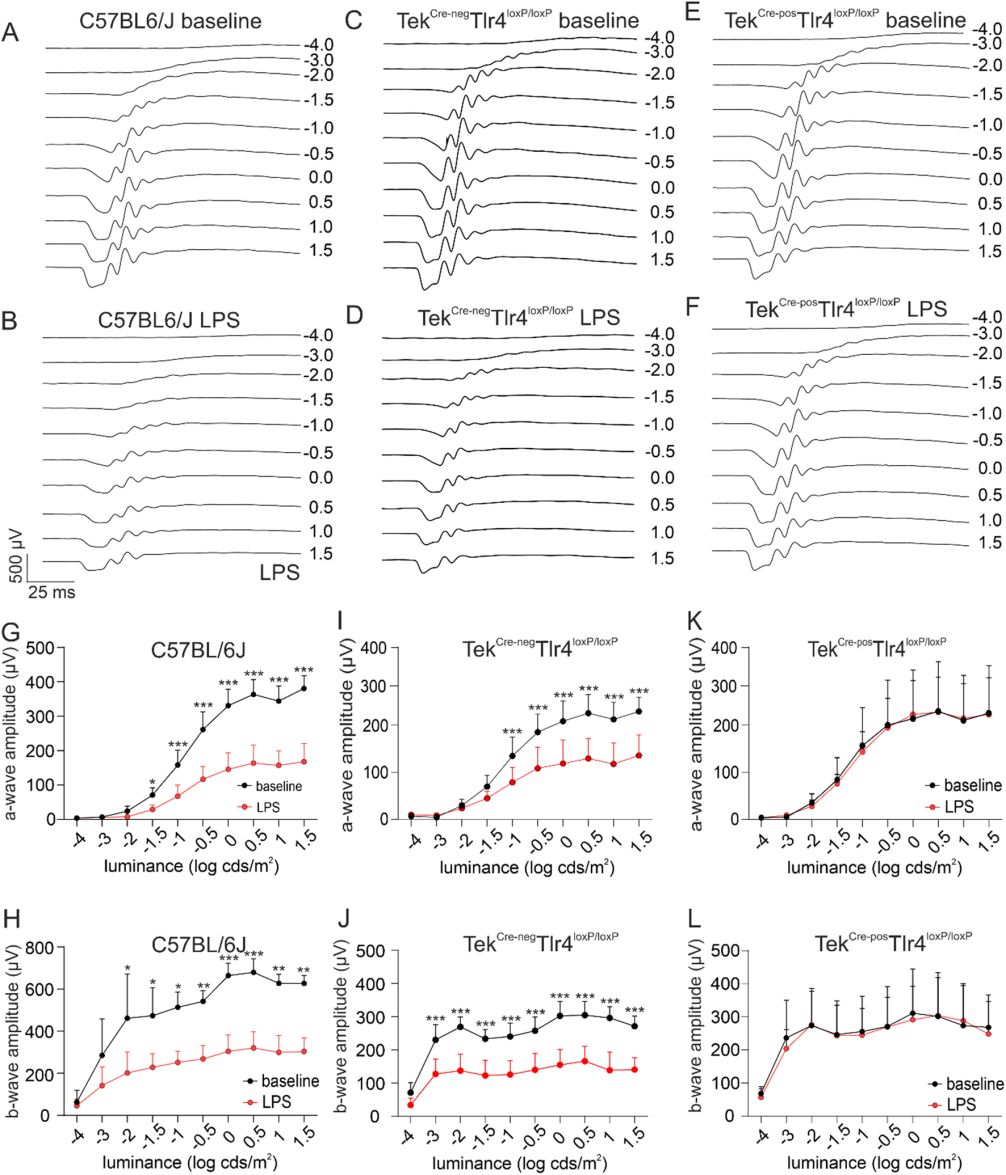
Endothelial *Tlr4* depletion rescue the retina from LPS-induced visual dysfunction. Representative waveforms at baseline (**A**, **C**, **E**) and after the LPS challenge (**B**, **D**, **F**) under scotopic conditions in C57BL/6J, Tek^Cre−neg^Tlr4^loxP/loxP^ and Tek^Cre−pos^Tlr4^loxP/loxP^ mice, respectively. Comparison of *a*- (**G**, **I**, **K**) and *b*-wave amplitudes (**H**, **J**, **L**) between baseline and LPS in C57BL/6J, Tek^Cre−neg^Tlr4^loxP/loxP^ and Tek^Cre−pos^Tlr4^loxP/loxP^ mice, respectively. Reduced mean *a*- and *b*-wave amplitudes were recorded in C57BL/6J and Tek^Cre−neg^Tlr4^loxP/loxP^ mice after the LPS challenge. In Tek^Cre−pos^Tlr4^loxP/loxP^ mice, mean *a*- and *b*-wave amplitudes after the LPS challenge were comparable to baseline levels. The data were analyzed with repeated measures 2-way ANOVA followed by Sidak’s post hoc analysis (*n* = 4–5 mice per genotype, one eye per mouse; *p < 0.05, **p < 0.01, ***p < 0.001)

**Fig. 5:**
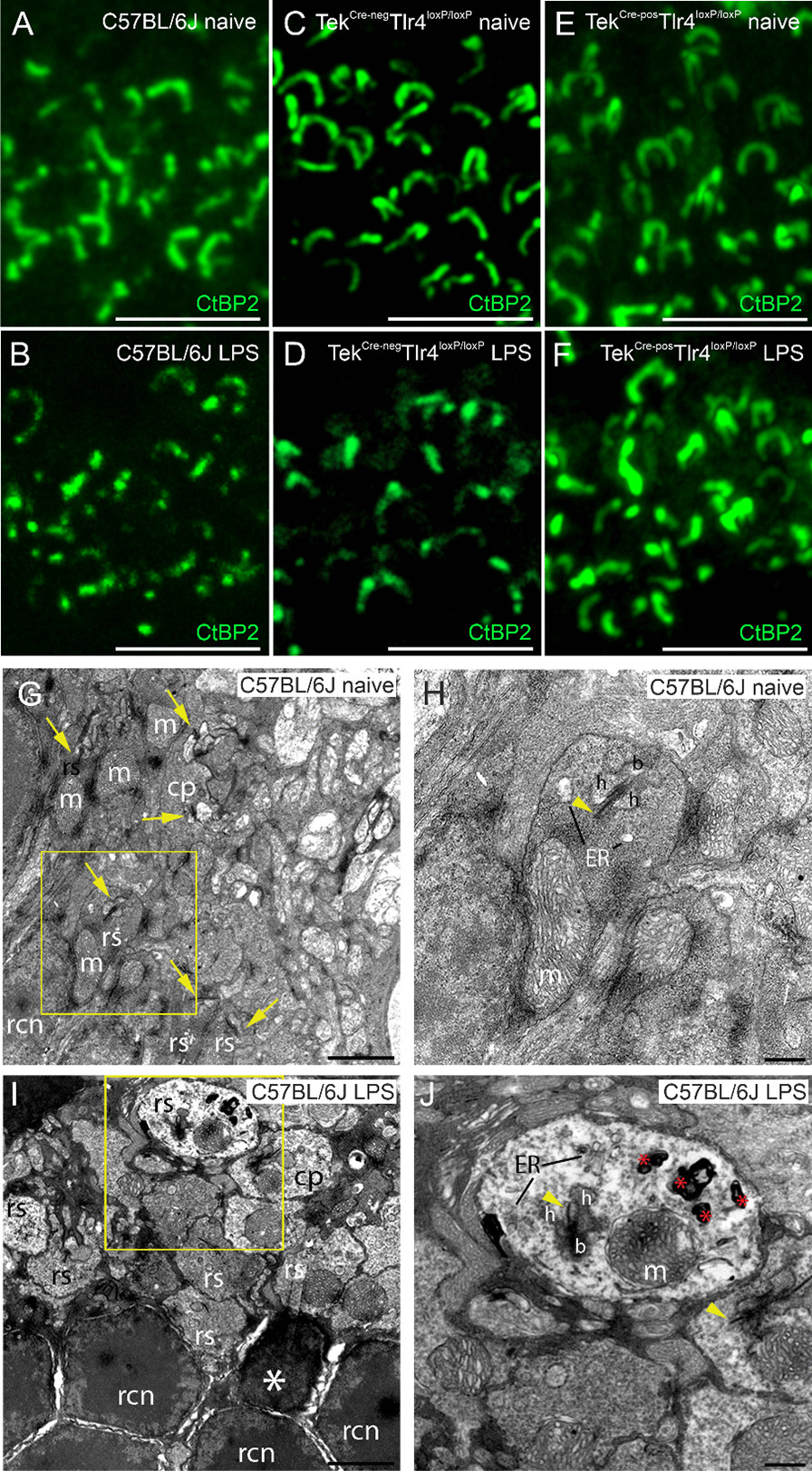
OPL ultrastructure in C57BL/6J mice is affected upon the LPS challenge. Representative z-stack images of retinal sections stained with the ribbon synapse marker CtBP2 in naïve (**A**, **C**, **E**) and LPS-challenged (**B**, **D**, **F**) C57BL/6J, Tek^Cre−neg^Tlr4^loxP/loxP^ and Tek^Cre−pos^Tlr4^loxP/loxP^ mice, respectively. In the LPS-challenged C57BL/6J and Tek^Cre−neg^Tlr4^loxP/loxP^ mice, ribbon synapses lose their characteristic shape and are less abundant in the OPL. Representative transmission electron microscopy images (×8200 magnification) obtained from naïve (**G**) and LPS-challenged (**I**) C57BL/6 J mice. Higher magnification (×20,500) of the yellow squares in G, I are shown in **H**, **J**, respectively. The LPS exposure led to alterations in the OPL ultrastructure characterized by membrane whorls, electro-lucent cytoplasm and decreased vesicle density in rod spherules. Yellow arrows and arrowheads indicate ribbon synapses and ribbons, respectively. White asterisk indicates an affected rod and red asterisks indicate membrane whorls. *b* Bipolar cell dendritic process; *cp* cone pedicle; *ER* endoplasmic reticulum; *h* horizontal cell axon tip; *m* mitochondrion; *rcn* rod cell nucleus; *rs* rod spherule. Scale bars: **A**–**F** 5 μm; **G**, **I** 2 μm. **H**, **J** 500 nm

To assess the effect of systemic LPS exposure on retinal function and the role of endothelial TLR4, full-field ERG responses were recorded at baseline and after the 4th LPS challenge in C57BL/6J, Tek^Cre−neg^Tlr4^loxP/loxP^ mice and Tek^Cre−pos^Tlr4^loxP/loxP^ mice (Fig. 4A–F). In C57BL/6J mice, under scotopic conditions, *a*- and *b*-wave amplitudes were decreased after the LPS challenge, and this became more pronounced at higher light intensities (Fig. 4G, H). Decrease in scotopic *a*- and *b*-wave amplitudes was also detected in the retinas of LPS-challenged Tek^Cre−neg^Tlr4^loxP/loxP^ mice (Fig. 4I, J). In the Tek^Cre−pos^Tlr4^loxP/loxP^ mice, no significant changes were observed between the baseline measurements and the measurements after the LPS challenge (Fig. 4K, L). However, in naïve Tek^Cre−neg^Tlr4^loxP/loxP^ and Tek^Cre−pos^Tlr4^loxP/loxP^ mice, lower mean *a*- and *b*-wave amplitudes were recorded compared to naïve C57BL/6J mice. No differences on *a*- and *b*-wave amplitudes were detected before and after the LPS challenge in photopic conditions for either genotypes (data not shown). Subsequently, immunohistochemistry against the presynaptic marker CtBP2 was employed. In the naïve retinas of C57BL/6J, Tek^Cre−neg^Tlr4^loxP/loxP^ and Tek^Cre−pos^Tlr4^loxP/loxP^ mice, CtBP2-expressing ribbon synapses localized in the OPL had a characteristic horseshoe shape (Fig. 5A, C, E). After the LPS challenge ribbon synapses lost their characteristic shape and they were less abundant in the OPL of C57BL/6J and Tek^Cre−neg^Tlr4^loxP/loxP^ mice (Fig. 5B, D), suggestive of synaptic impairment. No alteration on ribbon synapses shape or abundancy was detected in the Tek^Cre−pos^Tlr4^loxP/loxP^ retinas after the LPS exposure (Fig. 5F).

In parallel, TEM was performed in C57BL/6J retinas to investigate possible alterations on ribbon synapses ultrastructure in the presence of LPS. TEM revealed differences in the ultrastructure of rod spherules between naïve and LPS-challenged mice. Cone pedicles (cp) with multiple ribbon synapses and rod spherules (rs) with a single ribbon synapse could be visualized in the naïve and LPS-challenged retinas (Fig. 5G, I). The typical presentation of a ribbon synapse consists of the triad of two horizontal cell axon tips, a bipolar cell dendritic process, an arciform density, the presynaptic membrane and similar sized synaptic vesicles distributed across the spherule (Fig. 5H). In the LPS group, rod spherules and cone pedicles with less electron-dense cytoplasm were detected (Fig. 5I, J). A compressed rod photoreceptor cell body with condensed nucleus is shown in Fig. 5I (white asterisk). Membrane whorls were present in rod spherules with electro-lucent cytoplasm and decreased vesicle density (Fig. 5J, red asterisks).

## Discussion

The results of the present study demonstrate for the first time that activation of endothelial TLR4 is the first essential step for the sequel of events that lead to microglia activation, monocyte-derived macrophages infiltration into the retina and impaired retinal function upon systemic LPS exposure.

First, we generated mice that lack *Tlr4* expression selectively on endothelial cells to investigate the role of endothelial TLR4 in systemic LPS-induced retinal inflammation. To substantiate the validity of our conditional knockout mice, we utilized retinal whole mounts in Tek^Cre^tdTomato reporter mice and we confirmed that these mice express Cre recombinase selectively in endothelial cells and not Iba-1-positive cells (Fig. 1). Additionally, flow cytometry analysis substantiated TLR4 depletion in TIE2-expressing retinal endothelial cells in our Tek^Cre−pos^Tlr4^loxP/loxP^ mice (Fig. 1). The effect of repetitive systemic LPS exposures on the retina was investigated in C57BL/6J, Tek^Cre−neg^Tlr4^loxP/loxP^ and Tek^Cre−pos^Tlr4^loxP/loxP^ mice. Compared to our previous study in albino Balb/c mice; which are more susceptible to bacterial infection [50, 51]; here, we did not detect major disruption of the blood retinal barrier as examined by fluorescein angiography in C57BL/6J mice (Fig. 2). However, this method would only detect large disturbances based on fluorescein size (376.7 kDa; [52]). We did detect however, retinal vein dilation and monocyte-derived macrophages influx, which support BRB disruption (Figs. 2, 3). In agreement with our previous study and studies in the brain [16, 19], we observed that microglia from C57BL/6J and Tek^Cre−neg^Tlr4^loxP/loxP^ mice adopt an activated phenotype after the LPS challenge and they accumulate around retinal blood vessels (Fig. 2). At the same time, expression of ICAM-1 in the deep and intermediate vascular plexus correlated with elevated numbers of macrophages as shown by FACS analysis (Fig. 3). Interactions between ICAM-1 expressed by endothelial cells and leukocyte function-associated antigen-1 (LFA-1) expressed by leukocytes, contributes to the migration of leukocytes across the endothelium, partially through endothelial cell separation [53, 54].

Next, based on previous observations that systemic LPS exposure leads to permanent impairment of retinal function in P4 mouse pups [15], we performed ERG to detect possible LPS-induced functional impairments in the adult retina (Fig. 4). ERG showed reduced *a*- and *b*-wave amplitudes after the LPS exposure, reflecting the activity of photoreceptors and mainly ON bipolar cells, respectively. These effects were correlated with ribbon synapses damage as shown by immunohistochemistry and transmission electron microscopy (Fig. 5). Interestingly, similar impaired ERG responses and synapse dysfunction have been observed in microglia-depleted mice [55]. Engulfing and elimination of synapses by microglia during development is necessary for synapse maturation in the brain [56] and there is a hypothesis that microglia may influence synaptic function in the adulthood as well. In a rat model of retinitis pigmentosa, microglia cells were shown to phagocytize synaptic elements and this phagocytic activity was increased when synapses were destroyed [57]. In the present study, we did not observe internalization of CtBP2-positive elements from Iba-1^pos^ cells (Additional file 2: Video S1) indicating that microglia may not directly affect synapses in our model. Interestingly, retinal microglia despite sharing a common developmental lineage, seem to have different functions depending on their location in the retina, determined by interleukin 34 (IL-34) dependency. Specifically, IL-34-dependent microglia that reside in the IPL seem to play an important role in retinal function since IL-34-deficient mice have reduced numbers of microglia in the IPL and reduced *b*-wave responses, suggesting that they may play a role in visual processing [58]. Thus, we speculate that microglia is not responsible per se for synapse elimination upon the LPS challenge. In contrast, our data in combination with the studies mentioned above, suggest that given the role of microglia in homeostatic synaptic regulation, disruption of microglia–neuronal interactions due to the diversion of microglia activity towards the vasculature may account for the observed LPS-induced impaired retinal function as it has been suggested in the brain [59].

The results in our wild-type C57BL/6J mice correlate well with observations in the brain, where local LPS injection induced microglia and endothelium activation and recruitment of leukocytes from the periphery [60]. Based on their location in the vessel lumen, endothelial cells are of the first encounters of pathogens present in the circulation, and they can modulate subsequent inflammatory responses [61]. Additionally, leukocytes that circulate in the blood express TLR4 and can be directly affected by systemic LPS exposure. Systemic LPS exposure in mice that expressed TLR4 exclusively in endothelial cells led to neutrophil rolling and adhesion in brain vessels but failed to trigger their influx into the brain parenchyma. In the same study, expression of TLR4 by microglia rather than circulating cells was required for recruitment of neutrophils into the CNS. The authors suggested that direct activation of endothelial TLR4 by LPS is sufficient to initiate leukocyte–endothelial interactions, but TLR4 activation on brain microglia is required for the entry of leukocytes into the brain parenchyma [62]. The same group has previously reported that inhibition of microglia activation with minocycline did not affect the activation of endothelial cells in vitro [60], suggesting that microglia do not play a major role in endothelial activation.

Here, using conditional knockout mice, we show that in absence of endothelial TLR4, systemic LPS fails to induce any effect in the retina in terms of microglia activation, retinal vasodilation, monocyte-derived macrophages influx or synaptic impairment (Figs. 2, 3, 4, 5). A recent study showed that activation of microglia by systemic LPS is mediated by cytokines released from activated endothelial cells together with direct activation of TLR4 expressed by microglia in the perivascular space [20]. Activated endothelial cells release chemokines and cytokines, such as the chemokine (C–C motif) ligands 2, 7 and 20 (Ccl2, Ccl7 and Ccl20, respectively) [63] and inflammatory molecules, such as nitric oxide [64], which attract microglia to the affected vasculature in an attempt to maintain the integrity of the barrier. This involves expression of tight-junction proteins and establishment of physical contact with the endothelial cells [65]. Previous studies has shown that only a minimal amount of LPS (approximately 0.025% of the injected amount) can enter the brain parenchyma after intravenous LPS administration in mice, while the majority of LPS interacts with the BBB via binding to the luminal surface irreversibly [66]. We did not detect any vasodilation or signs of microglia/macrophages activation in the absence of endothelial TLR4 (Fig. 2), suggesting that the LPS-induced microglia activation that was observed in the present study depends on the activation of TLR4 located on endothelial cells. Moreover, we did not detect differences in the ERG responses of our conditional knockout mice before and after the LPS challenge (Fig. 4). However, there were signs of retinal dysfunction in both Cre-negative and Cre-positive Tlr4^loxP/loxP^ mice, revealed by lower ERG responses at baseline compared to C57BL/6J mice, suggesting that irrespective of Tlr4 deficiency in endothelial cells, the genetic background of these mice may have an effect on retinal function under normal conditions.

## Conclusions

**Fig. 6:**
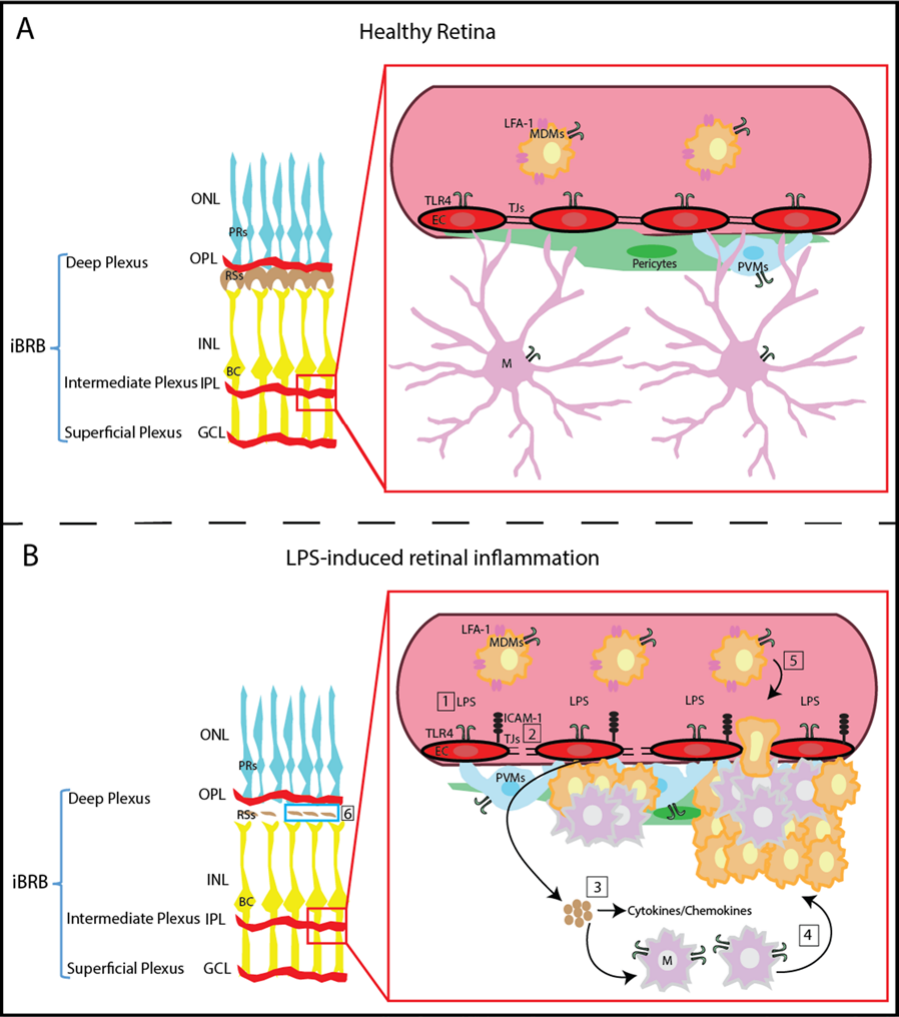
Proposed mechanism of systemic LPS-induced retinal microglia activation and retinal dysfunction. **A.** The inner BRB (iBRB) is composed by a deep, an intermediate, and a superficial vascular plexus. In the healthy retina, endothelial cells, that line the blood vessels, are connected by tight junctions forming an effective vascular barrier. Endothelial cells bear TLR4 receptors and they are surrounded by pericytes and perivascular macrophages. Microglia resides in close proximity to the vasculature, while monocytes circulate in the bloodstream. **B.** Upon the systemic LPS challenge, LPS binds to TLR4 located on circulating monocytes and endothelial cells (1), and induces the expression of the adhesion molecule ICAM-1 from the latter (2). Activated endothelial cells release cytokines and chemokines (3), which may act as chemoattractants causing microglia migration towards the affected vasculature (4). Subsequently, monocyte-derived macrophages are entering the retina through the disrupted BRB and AIF-1 – ICAM-1 interactions (5). Migration of microglia away from retinal neurons may account for disruption of ribbon synapses and impaired retinal function (6). *EC* endothelial cell; *GCL* ganglion cell layer; *iBRB* inner blood retinal barrier; *ICAM-1* intercellular molecule 1; *INL* inner nuclear layer; *IPL* inner plexiform layer; *LFA-1* lymphocyte function-associated antigen 1; *LPS* lipopolysaccharide; *M* microglia; *MDMs* monocyte-derived macrophages; *ONL* outer nuclear layer; *OPL* outer plexiform layer; *PRs* photoreceptors; *PVMs* perivascular macrophages; *RSs* ribbon synapses; *TJs* tight junctions; *TLR4* Toll-like receptor 4

Taken together, we hypothesize that after systemic LPS exposure the following series of events leads to retinal functional impairment (Fig. 6). First, retinal endothelial cells and circulating leukocytes are activated through binding of LPS to endothelial/leukocyte TLR4. Activated endothelial cells upregulate the expression of adhesion molecules, such as ICAM-1, and they release chemokines and cytokines that attract microglia towards the vasculature in an attempt to reintroduce homeostasis. Activated microglia are involved in the recruitment of monocyte-derived macrophages into the retina through the loosened BRB and these cells together with the loss of microglia–neuronal interaction may contribute to degeneration of ribbon synapses resulting in impaired retinal function. Overall, the present study provides evidence that endothelial TLR4 plays an essential role in retinal microglia activation following systemic LPS exposure and provides the ground for additional research about the endothelial cell–microglia/macrophages interactions and their possible role in retinal diseases with inflammatory component.

## Supplementary Information

**Additional file 1: Figure S1.** Gating strategy to identify microglia and macrophages in the retina. A population of interest was selected (**A**) and then cells were plotted based on forward scatter area (FSC-A) and high (FSC-H) for selection of single cells (**B**). CD3^neg^, CD19^neg^, NK1.1^neg^, Ly6G^neg^ and Zombie Green^neg^ cells were selected (**C**) and gated as CD11b^pos^ cells (**D**). In the CD11b^pos^ population, cells were gated as CD11b^pos^CD45^low/neg^ microglia and CD11b^pos^CD45^hi^ monocyte-derived macrophages (**E**).

**Additional file 2: Video S1.** Immunohistochemistry against Iba-1 and CtBP2 in LPS-challenged C57BL/6J mouse. Iba-1 is shown in magenta and CtBP2 in green. Scale bar: 20 μm.

## Data Availability

The data that support the findings of this study are available from the corresponding author upon reasonable request.

## Abbreviations

APC: Allophycocyanin
BBB: Blood brain barrier
BRB: Blood retinal barrier
CNS: Central nervous system
Ccl: Chemokine (C–C motif) ligand
cp: Cone pedicles
CtBP2: C-Terminal binding protein-2
ERG: Electroretinography
FA: Fluorescein angiography
FACS: Flow cytometry
FITC: Fluorescein isothiocyanate
GCL: Ganglion cell layer
Iba-1: Ionized calcium binding adapter molecule 1
ICAM-1: Intercellular adhesion molecule 1
IL-34: Interleukin-34
INL: Inner nuclear layer
IPL: Inner plexiform layer
LFA-1: Leukocyte function-associated antigen-1
LPS: Lipopolysaccharides
NGS: Normal goat serum
ONL: Outer nuclear layer
OPL: Outer plexiform layer
PE: Phycoerythrin
rs: Rod spherules
SD-OCT: Spectral-domain optical coherence tomography
TEM: Transmission electron microscopy
TRL: Toll-like receptor
