# Supplementary figures and images for: Endothelial Toll-like receptor 4 is required for microglia activation in the murine retina after systemic lipopolysaccharide exposure
Source: J Neuroinflammation. 2023 Feb 4;20:25. doi: 10.1186/s12974-023-02712-1 (PMC9899393; doi:10.1186/s12974-023-02712-1)

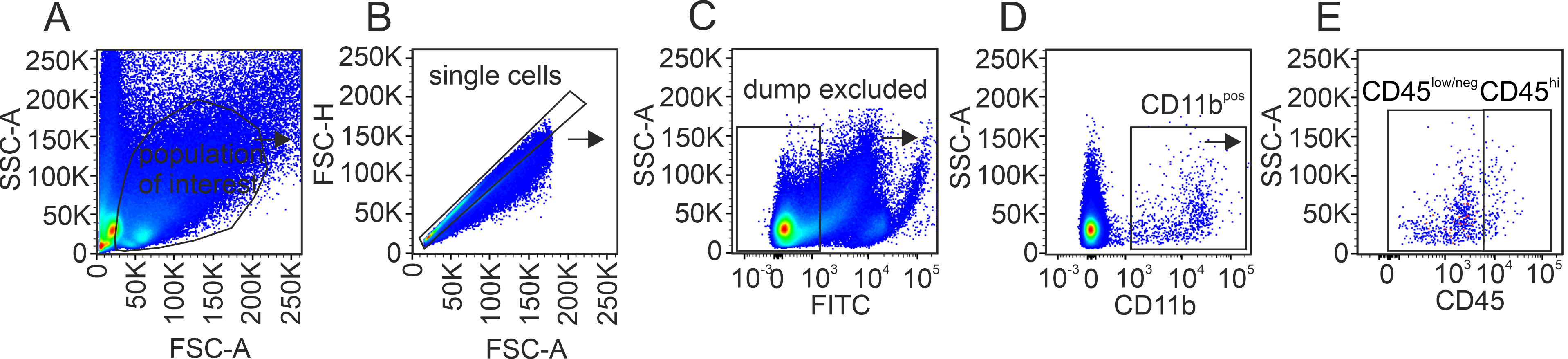

Supplement: Supplementary file 1 — Additional file 1: Figure S1. Gating strategy to identify microglia and macrophages in the retina. A population of interest was selected (A) and then cells were plotted based on forward scatter area (FSC-A) and high (FSC-H) for selection of single cells (B). CD3neg, CD19neg, NK1.1neg, Ly6Gneg and Zombie Greenneg cells were selected (C) and gated as CD11bpos cells (D). In the CD11bpos population, cells were gated as CD11bposCD45low/neg microglia and CD11bposCD45hi monocyte-derived macrophages (E). [file 12974_2023_2712_MOESM1_ESM.tif]
